# Fatigue Behavior of the FGH96 Superalloy under High-Temperature Cyclic Loading

**DOI:** 10.3390/ma16175883

**Published:** 2023-08-28

**Authors:** Zhengguang Li, Haiqin Qin, Kejun Xu, Zhenbo Xie, Pengcheng Ji, Mingming Jia

**Affiliations:** Qingdao Campus, Naval Aviation University, Qingdao 266041, China

**Keywords:** powder metallurgy superalloys, high-temperature low-cycle fatigue, creep–fatigue interaction, cyclic softening/hardening, ratcheting behavior, high-temperature oxidation

## Abstract

Strain-controlled low-cycle fatigue (LCF) tests and stress-controlled creep-fatigue interaction (CFI) tests on the FGH96 superalloy were carried out at 550 °C to obtain the cyclic softening/hardening characteristics at different strain amplitudes and ratcheting strain characteristics under different hold time. The failure mechanism of the FGH96 superalloy under different loading conditions was analyzed through fracture observations. The results show that the FGH96 superalloy exhibits different cyclic softening/hardening characteristics at different strain amplitudes, and the introduction of the hold time at peak stress exacerbates the ratcheting strain of the FGH96 superalloy under asymmetric stress cycles. Fracture observations show that the magnitude of the strain amplitude, high-temperature oxidation, and the introduction of the hold time will affect the mechanical properties of the FGH96 superalloy and change its fracture mode.

## 1. Introduction

With the continuous improvement of aero-engine performances, the turbine inlet temperature, speed, and engine thrust continue to increase, and the load conditions of the turbine system become increasingly harsh, which puts higher requirements on the mechanical properties of the key hot end parts of the aero-engine [1,2,3]. Due to its unique formation process, uniform structure, lack of macro segregation, high yield and tensile strengths, good creep resistance, and fatigue resistance, the powder metallurgy superalloy has become the material of choice for aero-engine turbine systems [4]. The FGH96 superalloy is a second-generation, damage-tolerant, nickel-based powder metallurgy superalloy from China. With its excellent high-temperature mechanical properties, the FGH96 superalloy has been widely used in key hot-end components, such as aero-engine high-pressure turbine disks [5,6]. Aero-engine high-pressure turbine disks are subjected to a large number of high-temperature cyclic loads when they are used [3], and the resulting high-temperature low-cycle fatigue (LCF) damage and creep-fatigue interaction (CFI) damage are the main damage forms that limit their longevity and reliability.

Many scholars have carried out related mechanical tests on the FGH96 superalloy. Hu et al. [7] studied the effect of inclusions on the LCF life of the FGH96 superalloy. Xiao et al. [8] carried out CFI experiments on FGH96 superalloy under different hold times and obtained qualitative properties of the cyclic softening of the FGH96 superalloy, but did not study the law of ratcheting evolution in depth. Zhang et al. [9] studied the influence of the microstructure on the fatigue crack growth behavior of FGH96 and proposed a new method for life prediction using relevant mechanical parameters. Miao et al. [10] used scanning electron microscopy to observe the fatigue fracture of FGH96 superalloy specimens under different stresses and analyzed the failure mechanism of the FGH96 superalloy high-temperature fatigue at the microstructure level. Li et al. [11] analyzed the influence of molten salt hot corrosion on the high-temperature and LCF behaviors of the FGH96 superalloy through experiments. Wang et al. [12] established a steady-state creep rate model for the FGH96 superalloy through creep experiments after pre-stretching it at room temperature. Shi et al. [13,14] artificially introduced defects on smooth samples of FGH96 superalloy and studied the effect of surface defects on the fatigue life of the FGH96 superalloy. Bai et al. [3] used synchrotron radiation X-ray imaging technology to test the fatigue performance of the FGH96 superalloy turbine disks to determine their fatigue life limit.

However, most of the aforementioned literature focuses on macroscopic qualitative research on the FGH96 superalloy or the LCF life, lacking studies on the cyclic soft/hardening law under cyclic strain loading conditions and the ratcheting strain behaviors of the CFI under typical asymmetric stress loading conditions. The above studies are very important for establishing the constitutive model of the FGH96 superalloy, life, and reliability analysis in engineering applications [15]. In order to achieve the reliable application of the FGH96 superalloy in the turbine structure of aero-engines, it is still necessary to carry out relevant systematic and in-depth research.

## 2. Experimental Materials and Methods

**Figure 1:**
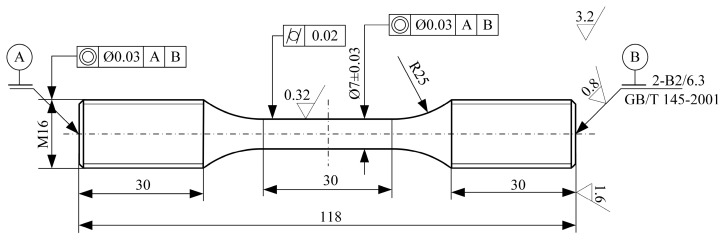
Shape and size of the FGH96 superalloy specimen (unit: mm) [16].

The specimens used in this study are FGH96 superalloy cylindrical samples. The two ends of the specimen are mounted in the testing machine by M16 thread. The diameter of the test section is 7 mm and the length of it is 30 mm. The chemical composition by measurement, shape, and size of the specimen are shown in Table 1 and Figure 1.

**Figure 2:**
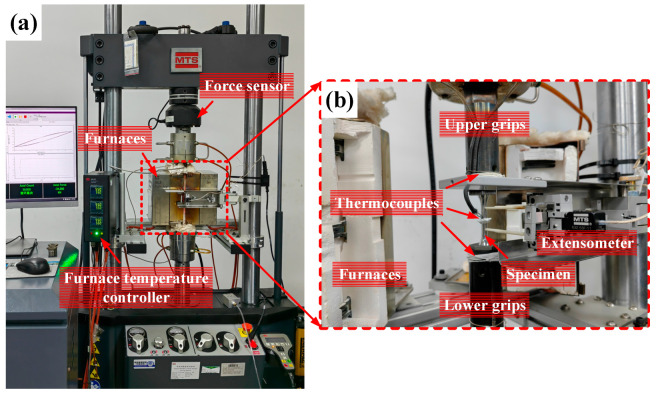
Test system: (**a**) testing machine; (**b**) specimen installation diagram.

The main work conducted in this paper is as follows: A series of strain-controlled LCF tests, stress-controlled LCF tests, and CFI tests were conducted by selecting specific temperatures and loads as the test conditions. According to the experimental results, the cyclic soft/hardening characteristics and ratcheting behavior of the FGH96 superalloy under high-temperature cyclic loading were summarized, and then the damage mechanism was analyzed through fracture observation. The specific details of the experiment are as follows:

All tests were carried out using an electro-hydraulic servo fatigue testing machine (MTS Systems Co., MTS-370.1, Eden Prairie, MN, USA) with a maximum load capacity of 100 kN equipped with high-temperature thermal chamber, and deformation was measured using an axial high-temperature extensometer (MTS Systems Co., MTS 632.53F-11, USA) with a gauge length of 25 mm. The maximum operating temperature of the turbine disk life assessment point for a certain type of aero-engine is 519 °C. Considering the fluctuation of temperature and the error of the sensor during the actual flight, the temperature of all the tests was set at 550 °C for conservative reasons. The temperature was measured using K-type thermocouples in the upper, middle, and lower sections, and the high-temperature furnace (MTS Systems Co., MTS 653, USA) was used for heating, and the tests started after the temperature was stable for 30 min. The test system is shown in Figure 2.

**Figure 3:**
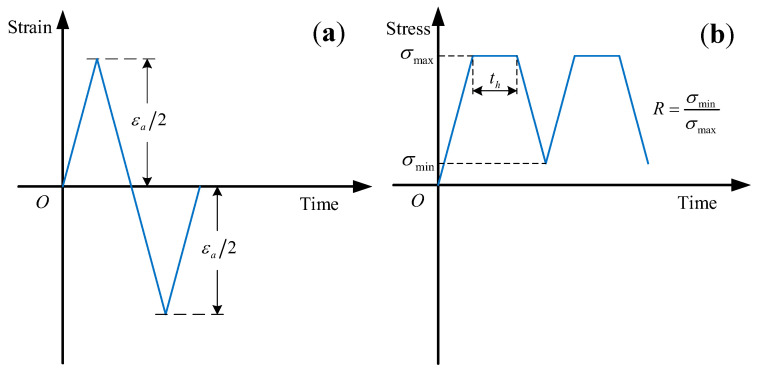
Test loading waveform: (**a**) strain-controlled cyclic tests; (**b**) stress-controlled cyclic tests.

In order to test the cyclic softening/hardening characteristics of the FGH96 superalloy at a high temperature, the cyclic strain-controlled cyclic loading in the symmetric tension-compression direction was selected for strain cyclic tests. Considering that the turbine disk is mainly subjected to cyclic loading in the tension–tension direction in actual work, according to the actual load condition of the aero-engine turbine disk, this direction was selected for stress-controlled cyclic tests. The hold time was introduced at peak stress to simulate the steady-state condition. The specific method and scheme of the experiment are as follows:

(1) Strain-controlled cyclic tests: The test method refers to GB/T 26077-2021 “Metallic materials—Fatigue testing—Axial-strain-controlled method” [17], and we selected the strain amplitudes $\varepsilon_{a}/2$ = 0.5%, 0.6%, 0.8%, and 1.2% for the strain-controlled cyclic tests. The test waveform adopts a symmetrical triangular waveform with a strain loading/unloading rate of 0.005 s^-1^ and a strain ratio *R* = −1, as shown in Figure 3a.

(2) Stress-controlled cyclic tests: We selected the typical working condition 1285 MPa as the stress peak value for testing, which corresponded to the life assessment point of the high-pressure turbine disk of this type of engine. The test method refers to GB/T 3075-2021 “Metallic materials—Fatigue testing—Axial force-controlled method” [18]. In order to simulate the steady-state situation in the actual working condition of the turbine disk, the hold time was introduced at the peak stress to carry out CFI tests. The test method refers to GB/T 38822-2020 “Metallic materials—Creep–fatigue test method” [19]. We selected the following hold time: $t_{h}$ = 30 s, 60 s. The test waveform adopts an asymmetric trapezoidal waveform with a stress loading/unloading rate of 500 MPa/s and a stress ratio *R* = 0.06, as shown in Figure 3b.

**Figure 4:**
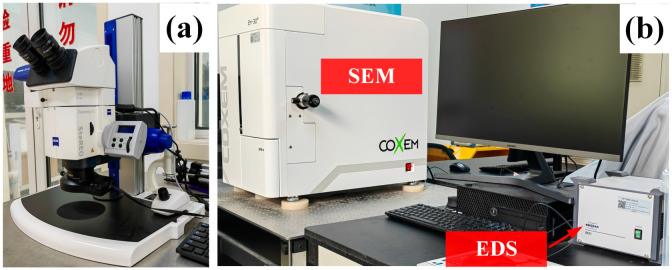
Fracture analysis system: (**a**) optical microscope (OM); (**b**) scanning electron microscope (SEM) and energy dispersive spectrometer (EDS).

After the cyclic tests, an optical microscope (OM, Carl Zeiss AG SteREO Discovery.V12, Oberkochen, Germany), scanning electron microscope (SEM, COXEM EM-30^+^, Daejeon, Republic of Korea), and energy dispersive spectrometer (EDS, Bruker Nano GmbH, Berlin, Germany) were used to observe and analyze the fracture features of the specimens. Among them, EDS was connected to SEM, which was used to observe the element composition of specific areas. The related equipment is shown in Figure 4.

## 3. Results and Discussion

### 3.1. Strain-Controlled Cyclic Tests

In order to explore the cyclic softening/hardening characteristics of FGH96 superalloy in a high-temperature environment, uniaxially symmetrical strain-controlled cyclic tests were carried out at 550 °C.

**Figure 5:**
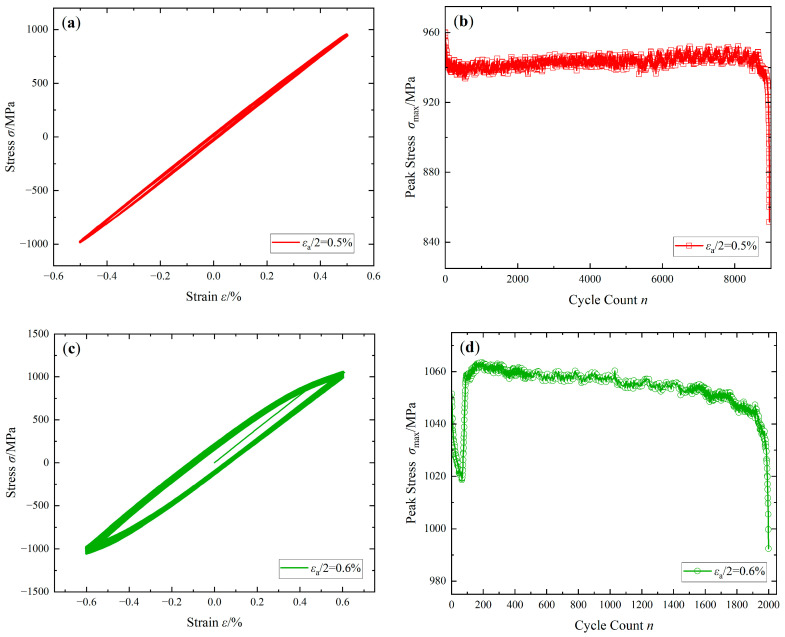

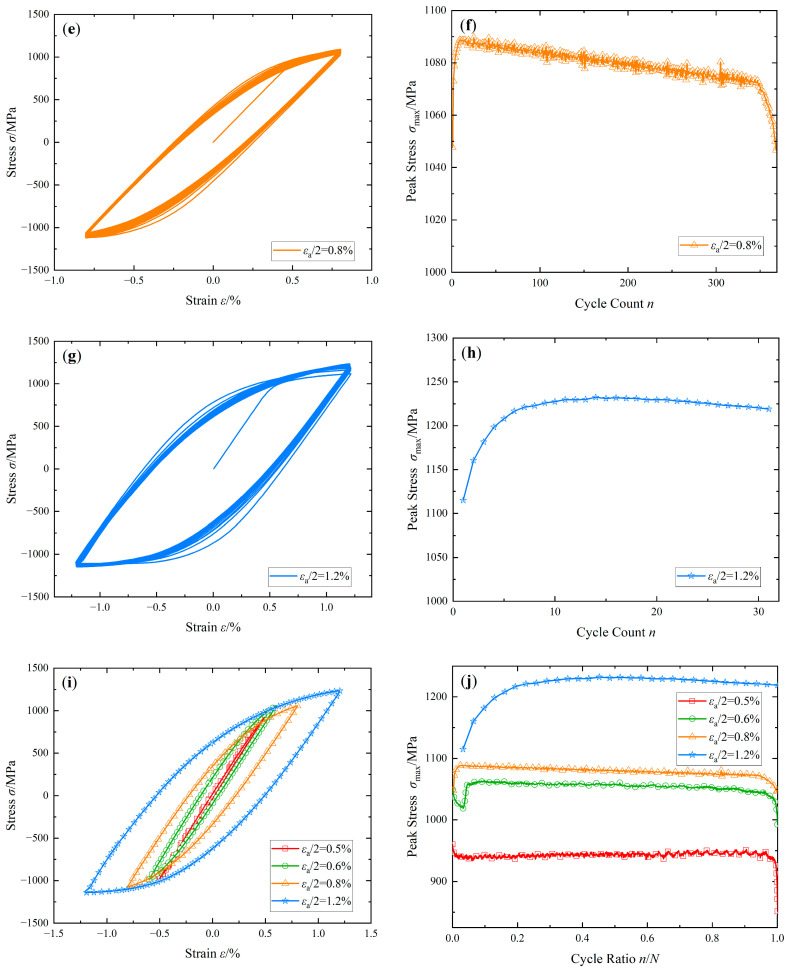
Strain-controlled cyclic tests curves: (**a**,**c**,**e**,**g**) stress-strain curves of $\varepsilon_{a}/2$ = 0.5%, 0.6%, 0.8%, and 1.2%; (**b**,**d**,**f**,**h**) peak stress-cycle count curves of $\varepsilon_{a}/2$ = 0.5%, 0.6%, 0.8%, and 1.2%; (**i**) half-life hysteresis loops; (**j**) peak stress-cycle ratio curves.

Figure 5a,c,e,g show full stress–strain curves with strain amplitudes $\varepsilon_{a}/2$ = 0.5%, 0.6%, 0.8%, and 1.2% respectively. Figure 5i shows half-life hysteresis loops under the above four strain amplitudes. Through comparison, it can be concluded that when the strain amplitude is low, the steady-state hysteresis loop is narrow and long, and the area is small; as the strain amplitude increases, the steady-state hysteresis loop becomes “full”, and the area gradually increases. The thickness of the whole hysteresis loop in Figure 5a,c,e,g reflects the overall trend of the material cyclic softening/hardening to a certain extent. In order to accurately analyze its change, the peak stress in each cycle was selected for observation.

Figure 5b,d,f,h show the relationship between the peak stress and the corresponding cycle number under the control of four strain amplitudes in each cycle. It can be found that under different strain amplitudes, the cyclic softening/hardening of the FGH96 superalloy shows different characteristics. At the strain amplitude $\varepsilon_{a}/2$ = 0.5%, the FGH96 superalloy first enters the cyclic softening stage, then enters the cyclic stability stage, and finally, undergoes cyclic softening until it fractures. At the strain amplitude $\varepsilon_{a}/2$ = 0.6%, the FGH96 superalloy is unstable during the early stage of the cycles (softening greatly first, and then hardening rapidly), and then stable in the cycle (with a tendency to soften slowly), it softens rapidly until it fractures at last. At the strain amplitude $\varepsilon_{a}/2$ = 0.8%, the FGH96 superalloy first undergoes cyclic hardening, and then cyclic softening until it fractures. At the strain amplitude $\varepsilon_{a}/2$ = 1.2%, the FGH96 superalloy undergoes cyclic hardening gradually, and when the cyclic hardening value reaches the stress saturation value, it undergoes slow cyclic softening until it fractures.

Figure 5j reflects the change in the peak stress corresponding to each cycle ratio n/N under the four strain amplitudes in the same coordinates, that is, the cycle softening/hardening situation. On the whole, during the early strain cycles, the softening/hardening conditions of the FGH96 superalloy under different strain amplitudes are quite different. At the strain amplitude $\varepsilon_{a}/2$ < 0.6%, the cyclic softening is dominant at the early stage of the cycles; at the amplitude $\varepsilon_{a}/2$ > 0.8%, the cyclic hardening is dominant at the early stage of the cycles; in the final cycles, the FGH96 superalloy mainly shows the property of cyclic softening.

### 3.2. Stress-Controlled Cyclic Tests

**Figure 6:**
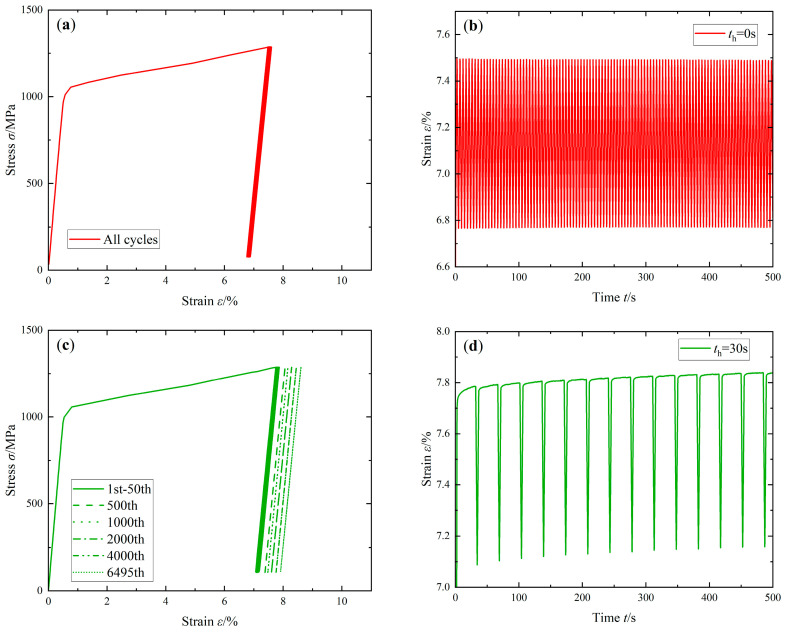

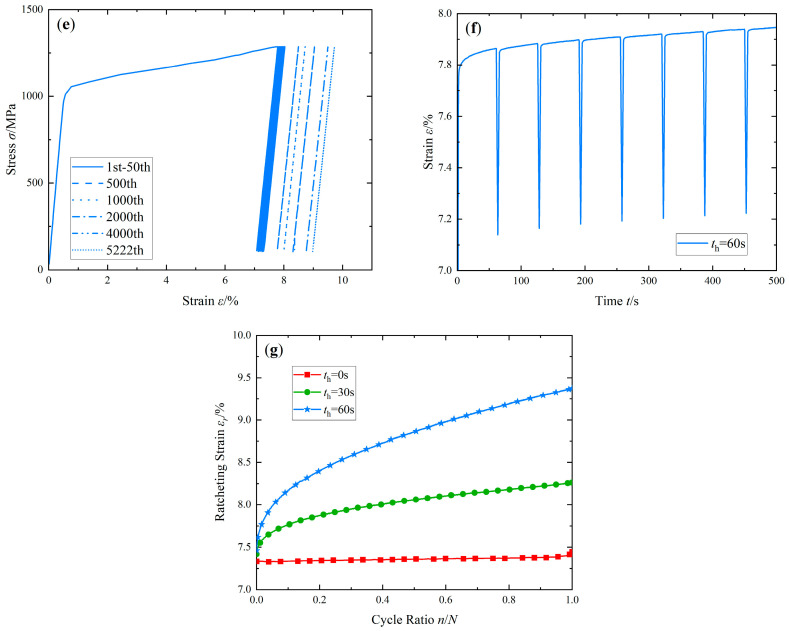
Stress-controlled cyclic tests curves under $\sigma_{\max}$ = 1285 MPa: (**a**,**c**,**e**) stress-strain curves of $t_{h}$ = 0 s, 30 s, and 60 s; (**b**,**d**,**f**) strain curves in the first 500 s of $t_{h}$ = 0 s, 30 s, and 60 s; (**g**) ratcheting strain-cycle ratio curves.

The stress-controlled cyclic tests were conducted in uniaxial asymmetric stress conditions. Figure 6a,c,e show the cyclic stress–strain curves at $t_{h}$ = 0 s, 30 s, and 60 s under the stress peak $\sigma_{\max}$ = 1285 MPa. Through this observation, it can be found that regardless of the length of the holding time, the stress-strain curve under a single stress cycle is very narrow and can be approximated as a straight line, and no obvious hysteresis loop is formed. It can be seen from the comparison that with the increase in the hold time, a larger strain value is produced under the same number of cycles. Figure 6b,d,f show the curves of strain with time at $t_{h}$ = 0 s, 30 s, and 60 s in the first 500 s of the tests. It can be seen in Figure 6b that the strain value of the FGH96 superalloy does not change significantly with the increase in the number of cycles at $t_{h}$ = 0 s. After introducing the hold time at the peak stress, due to the creep effect at a high temperature, the FGH96 superalloy produces an obvious stress relaxation phenomenon under each stress cycle. When the stress is unloaded and reloaded, although the strain is slightly lower than that before unloading, the strain rate still develops and accumulates along the rate before unloading (Figure 6d,f).

In order to compare the changes in the ratcheting effect, Figure 6g shows the change curve of the ratcheting strain with the cycle ratio under different hold times. In this paper, the ratcheting strain $\varepsilon_{r}$ is defined as the mean value of the maximum strain $\varepsilon_{\max}$ and the minimum strain $\varepsilon_{\min}$ in each cycle, that is, $\varepsilon_{r}=\left(\varepsilon_{\max}+\varepsilon_{\min}\right)/2$. It can be seen from the comparison that the impact of the hold time on the ratcheting strain of the FGH96 superalloy is very obvious. At the hold time $t_{h}$ = 0 s, the ratcheting behavior of the FGH96 superalloy is not obvious, and the ratcheting strain only increases suddenly at the end of the cycle until it breaks. After peak stress is introduced during the hold time, which is affected by the CFI, the stress relaxation phenomenon occurs, and the ratcheting deformation of the FGH96 superalloy is significantly increased. At the same time, with the increase in the hold time, the ratcheting strain of the FGH96 superalloy increases more.

### 3.3. Fracture Features

In order to deeply understand the damage mechanism of the FGH96 superalloy under high-temperature cyclic loading, the fracture of the specimens was observed and analyzed via OM and SEM.

**Figure 7:**
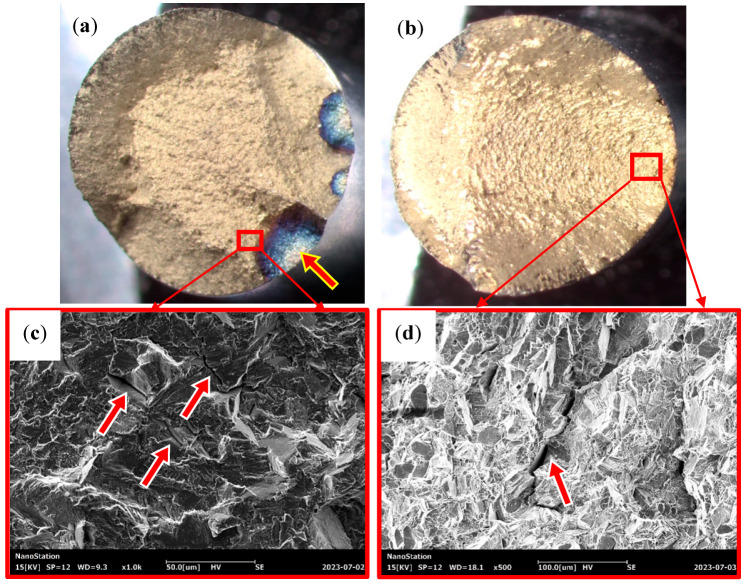
Fracture morphology of strain-controlled cyclic tests: (**a**,**c**) $\varepsilon_{a}/2$ = 0.6%; (**b**,**d**) $\varepsilon_{a}/2$ = 1.2%. The arrow in (**a**) indicates the position of EDS, the arrows in (**c**,**d**) indicate the position of the secondary cracks.

**Figure 8:**
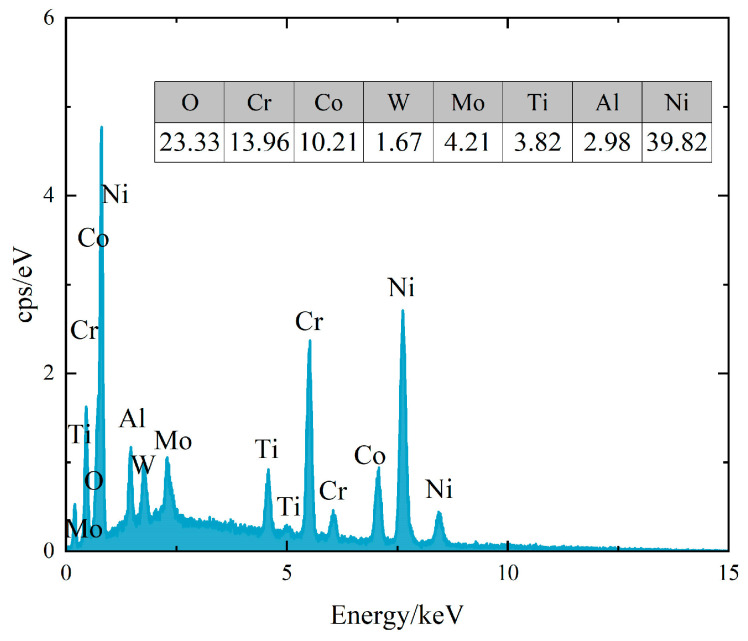
The EDS results of the crack source area (the table in the figure shows the elements contained in the analysis, wt./%).

Figure 7a,b show the macroscopic morphology of the fracture of the specimens when the strain amplitude $\varepsilon_{a}/2$ = 0.6% and 1.2%. Through this observation, it can be found that the cracks of the specimens under strain-controlled tests all formed on the surface of the specimens. The fracture is divided into an obvious crack source area, crack propagation area, and transient break area (shear lip morphology). At the strain amplitude $\varepsilon_{a}/2$ = 0.6%, multiple crack sources are observed on the fracture surface, and the crack source area is blue. At the strain amplitude $\varepsilon_{a}/2$ = 1.2%, only one source of the crack can be observed on the fracture surface. We used EDS to analyze the crack source area shown in Figure 7a (the analysis location is the area shown by the red arrow), and the analysis results are shown in Figure 8. It can be found that the crack source area has more oxygen elements, indicating that the crack source area is blue due to high-temperature oxidation.

In contrast to the law that states that the number of crack sources in the material fracture increases with the increase in the strain amplitude during the LCF test at room temperature [20], the number of crack sources in the FGH96 superalloy decreases with the increase in strain amplitude at a high temperature, which may be caused by high-temperature oxidation. Chen et al. [21] pointed out in their research on the nickel-based superalloy GH4169 that oxides on the surface of the specimen may fall off under cyclic loading, thus causing defects on the surface of the specimen, resulting in stress concentration and crack initiation. It can be inferred that the formation of cracks in the tests is closely related to high-temperature oxidation. Considering that the fatigue life at a low-strain amplitude is longer than that at a high-strain amplitude, those at a low-strain amplitude will be oxidized for a longer time, and the damage caused by high-temperature oxidation increases with the decrease in the strain amplitude. Therefore, at low-strain amplitudes, high-temperature oxidation initiates more crack sources. Li et al. [22] also found a similar rule in the isothermal fatigue test of 316LN stainless steel under different strain amplitudes. In addition, it can be observed that there is a large number of fatigue shell pattern lines in the crack propagation area at the strain amplitude $\varepsilon_{a}/2$ = 1.2%, indicating that this area bears more overload, reflecting the cyclic hardening phenomenon of the FGH96 superalloy under this strain amplitude.

Figure 7c,d show microscopic images corresponding to the crack propagation area. The comparison shows that there are more secondary cracks in the crack propagation zone at the strain amplitude $\varepsilon_{a}/2$ = 0.6%. However, the size is relatively small. A large number of fatigue stripes can be observed at the same time (Figure 7c). At the strain amplitude $\varepsilon_{a}/2$ = 1.2%, the secondary cracks observed are longer and deeper, but the fatigue bands are relatively inconspicuous (Figure 7d).

The above phenomenon shows the difference in microstructure damage characteristics of the FGH96 superalloy under different strain amplitudes. Recent studies have shown that the cyclic soft/hardening properties of metallic materials are closely related to the accumulation of inelastic strains and dislocation motion [22,23,24,25,26]. At the early stage of the cycle, the increase in strain amplitude accelerates the accumulation of inelastic strain in the material and intensifies the deterioration of the microstructure, which is manifested in the lengthening and deepening of the secondary cracks. The deterioration of the microstructure also causes an increase in the resistance to dislocation motion, leading to the strengthening of the material crystals, which manifests itself in the cyclic hardening behavior of the material. The larger the strain amplitude, the more obvious the cyclic hardening is. In the final cycles, with the continuous merging and expansion of the cracks, the degradation of material mechanical properties caused the FGH96 superalloy cyclic softening.

**Figure 9:**
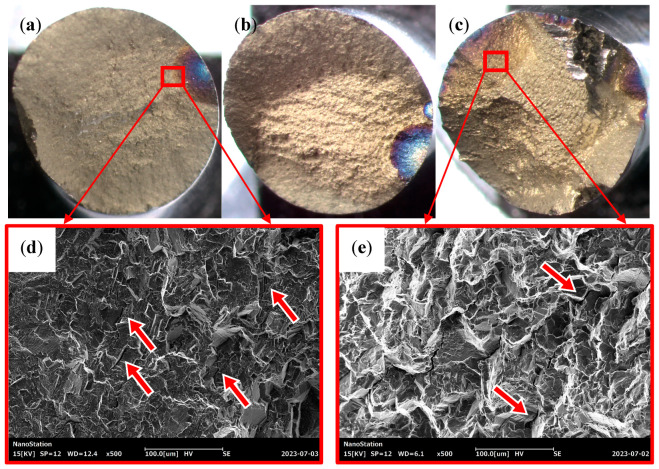
Fracture morphology of stress-controlled cyclic tests under $\sigma_{\max}$ = 1285 MPa: (**a**,**d**) $t_{h}$ = 0 s; (**b**) $t_{h}$ = 30 s; (**c**,**e**) $t_{h}$ = 60 s. The arrows in (**d**) indicate the position of transgranular cracks, the arrows in (**e**) indicate the position of intergranular cracks.

Figure 9a–c show the macroscopic fracture of the specimens at $t_{h}$ = 0 s, 30 s, and 60 s under $\sigma_{\max}$ = 1285 MPa. It can be observed that all fracture cracks originate from the blue oxidized area on the surface of the specimens and propagate from here to the interior of the material. Only one crack source region can be observed on the fracture without a hold time, and the fracture is relatively flat (Figure 9a). Multiple crack sources can be found for the fracture when the hold time was introduced at peak stress, and with the increase in the hold time, the number of crack sources also increases, and the fracture surface becomes rougher (Figure 9b,c).

Through the macroscopic observation of the fracture, it can be seen that an oxidation reaction also occurs on the surface of the FGH96 superalloy in a high-temperature environment. From the formation law of the crack source in the strain cycle test above, it can be inferred that the FGH96 superalloy also forms a crack source under cyclic loading due to oxidation, and this expands on the inside of the FGH96 superalloy specimen. At the same time, under the effect of creep in the tensile direction, the high-temperature oxidation time is prolonged, which intensifies the oxidation damage on the surface of the material and causes more crack sources to be generated under the joint damage of creep and high-temperature oxidation. Due to the simultaneous inward expansion of multiple crack sources at different locations, the fracture height difference of the specimen increases, and the section becomes rougher.

Figure 9d,e show the microscopic morphology of the crack propagation zone corresponding to $t_{h}$ = 0 s and 60 s; a large number of transgranular cracks can be observed in the crack propagation area of the specimen without a hold time (Figure 9d). In addition to transgranular cracks, more intergranular cracks (Figure 9e) are found in fractures with a hold time. Considering that the creep strain generated during the holding time will induce the nucleation and growth of vacancies at the grain boundaries, oxygen at a high temperature tends to form brittle oxides at the grain boundaries with higher diffusivity. Therefore, under the joint action of creep and oxidative damage, the initiation and propagation of intergranular cracks are promoted, and the fracture mode of the FGH96 superalloy has changed from transgranular to transgranular–intergranular.

## 4. Conclusions

In this paper, in order to study the mechanical properties of the FGH96 superalloy under high-temperature cyclic loading, a series of strain-controlled LCF tests and stress-controlled LCF tests with/without a hold time were carried out at 550 °C. The characteristics of the appearance were analyzed, and the conclusions are as follows:

(1) Under different strain amplitudes, the FGH96 superalloy exhibits different cyclic softening/hardening characteristics. When the amplitude is less than 0.6%, cyclic softening mainly occurs at the early stage of the cycles, and when the amplitude is greater than 0.8%, hardening mainly occurs at the early stage of the cycles. At the end of the cycles, the FGH96 superalloy mainly exhibits the characteristics of cyclic softening.

(2) Asymmetric stress-controlled cyclic loading in the tension–tension direction does not produce an obvious ratcheting effect on the FGH96 superalloy; when the hold time is introduced at peak stress, the CFI significantly increases the ratcheting strain of the FGH96 superalloy.

(3) An oxidation reaction occurs on the surface of the FGH96 superalloy specimen under cyclic loading at 550 °C and then forms a crack source, and a crack develops on the inside of the FGH96 superalloy until it breaks; the longer high-temperature cyclic loading continues for, the greater the number of crack sources on the surface of the specimen that are initiated.

(4) The fracture mode of the FGH96 superalloy gradually changes from transgranular fracture to transgranular–intergranular when the hold time is introduced at peak stress during stress-controlled cyclic loading.

## Figures and Tables

**Table 1:** Chemical composition of the FGH96 specimen (wt./%).

Element	Cr	Co	W	Mo	Ti	Al	Ni
wt./%	15.43	12.94	5.22	4.11	3.51	2.49	56.30

## Data Availability

The data presented in this study are available on request from the corresponding author.
